# Supplementary material for: Design and approval of the nutritional warnings’ policy in Peru: Milestones, key stakeholders, and policy drivers for its approval
Source: PLOS Glob Public Health. 2023 Jun 16;3(6):e0001121. doi: 10.1371/journal.pgph.0001121 (PMC10275441; doi:10.1371/journal.pgph.0001121)
Supplement: S1 Text — (DOCX) [file pgph.0001121.s001.docx]

**Supporting information 1**

**Code book**

(K) = Codes aligned with the Kaleidoscope Model’s variables

1. **Law**
   1. (K) Champions during the Design
   2. (K) Purpose of having nutritional warnings and/or designing a Law that includes the nutritional warnings
   3. Documents (Law, bills)
      1. Bills (2012)
      2. Dictum (2013)
      3. Approved Law (2013)
      4. (K) Studies/reports conducted or used during the Law’s design
      5. Experiences of warnings or legislation in other countries
   4. Descriptions and opinions about the Law
      1. About the parameters
      2. About the nutritional warnings
   5. Stakeholders’ postures/actions during the design and approval of the Law
      1. Food industry’s postures/actions
      2. Media (TV, newspapers, journalists)’s postures/actions
      3. Civil society organizations’ postures/actions
      4. Congress’s postures/actions
      5. Ministry of Health’s postures/actions
      6. Other government stakeholders’ postures/actions
      7. PAHO/WHO’s postures/actions
      8. Other stakeholders’ postures/actions
   6. Difficulties for the design and/or approval of the Law
      1. Specific difficulties: Lack of evidence
      2. Specific difficulties: Power of the food industry
      3. Specific difficulties: Power of opponents in the government
      4. Specific difficulties: Power of other opponents
   7. Enablers: Facts/actions that helped the Law to be designed and/or approved
      1. (K) Non-planned enablers. Facts that positioned the Law and nutritional warnings
      2. Intentional enablers. Actions/decisions taken so that the Law could be designed and/or approved
         1. Intentional enablers. Actions/decisions that worked/were helpful
   8. Recommendations or ideas that could enhance the Healthy Eating Law
      1. Explicit recommendations
      2. Proposals that got lost along the way
      3. What could have been done differently
      4. Critics
   9. General assessments of the Law
   10. Recommendations for other future policies (other than the Law and the octagons)
   11. Law. Other topics
2. **Regulation of the Law**
   1. (K) Purpose of having a Regulation
   2. Documents (Regulation, drafts)
      1. First Regulation’s draft (2014)
      2. Approval of parameters for sugar, sodium, and saturated fats (2015)
      3. Second Regulation’s draft (2016)
      4. Approval of parameters for trans fat (2016)
      5. Approved Regulation (2017)
      6. (K) Studies/reports conducted or used during the Regulation’s design
   3. Descriptions and opinions about the Regulation and its drafts
      1. About the parameters of the First Regulation’s Draft (Local data)
      2. About the parameters of the Second Regulation’s Draft (PAHO)
      3. About the parameters of the approved Regulation (Chilean)
   4. Stakeholders’ postures/actions during the design of the second Regulation’s draft
      1. Food industry’s postures/actions
      2. Civil society organizations’ postures/actions
      3. Ministry of Health’s postures/actions
      4. Other government stakeholders’ postures/actions
      5. PAHO/WHO’s postures/actions
      6. Other stakeholders’ postures/actions
   5. Stakeholders’ postures/actions during the design of the approved Regulation
      1. Food industry’s postures/actions
      2. Civil society organizations’ postures/actions
      3. Ministry of Health’s postures/actions
      4. Other government stakeholders’ postures/actions
      5. PAHO/WHO’s postures/actions
      6. Other stakeholders’ postures/actions
   6. Difficulties for the design and/or approval of the Regulation
      1. Specific difficulties: Lack of evidence
      2. Specific difficulties: Power of the food industry
      3. Specific difficulties: Power of opponents in the government
      4. Specific difficulties: Power of other opponents
   7. Enablers: Facts/actions that helped the Regulation to be designed and/or approved
      1. (K) Non-planned enablers. Facts that positioned the Regulation
      2. Intentional enablers. Actions/decisions taken so that the Regulation could be designed and/or approved
         1. Intentional enablers. Actions/decisions that worked/were helpful
   8. Recommendations or ideas that could enhance the Regulation
      1. Explicit recommendations
      2. Proposals that got lost along the way
      3. What could have been done differently
      4. Critics
   9. General assessments of the Regulation
      1. Reasons for the delay between the Law and the Regulation
   10. Regulation: Other topics
3. **Nutritional Warnings’ Manual**
   1. (K) Purpose of having the Manual
   2. Documents (Manual, drafts)
      1. Manual’s draft (2017)
      2. Approved Manual (2018)
      3. (K) Studies/reports conducted or used during the Manual’s design
   3. Descriptions and opinions about the Manual and its draft
   4. Stakeholders’ postures/actions during the design of the Manual’s draft
      1. Ministry of Health’s postures/actions
      2. Other stakeholders’ postures/actions
   5. Stakeholders’ postures/actions during the design of the approved Manual
      1. Food industry’s postures/actions
      2. Civil society organizations’ postures/actions
      3. Ministry of Health’s postures/actions
      4. Other government stakeholders’ postures/actions
      5. Other stakeholders’ postures/actions
   6. Difficulties for the design and/or approval of the Manual
      1. Specific difficulties: Lack of evidence
      2. Specific difficulties: Power of the food industry
      3. Specific difficulties: Power of opponents in the government
      4. Specific difficulties: Power of other opponents
   7. Enablers: Facts/actions that helped the Manual to be designed and/or approved
      1. (K) Non-planned enablers. Facts that positioned the Manual
      2. Intentional enablers. Actions/decisions taken so that the Manual could be designed and/or approved
         1. Intentional enablers. Actions/decisions that worked/were helpful
   8. Recommendations or ideas that could enhance the Manual
      1. Explicit recommendations
      2. Proposals that got lost along the way
      3. What could have been done differently
      4. Critics
   9. General assessments of the Manual
      1. Assessments of the octagons as a nutritional warning model
         1. Reasons why it was decided to use octagons
      2. Assessments of other warnings’ designs (Traffic light, GDA)
   10. Manual: Other topics
4. **Bills to amend the Law (and Regulation)**
   1. Postures/actions of stakeholders in favor of the Law
   2. Postures/actions of opponents of the Law
   3. Power of opponents of the Law
   4. Enablers: Facts/actions that helped that the bills did not prosper

**5. Comprehensive evaluations of all the documents**

**6. Comprehensive evaluations of all the stakeholders**
