# Supplementary material for: Design and approval of the nutritional warnings’ policy in Peru: Milestones, key stakeholders, and policy drivers for its approval
Source: PLOS Glob Public Health. 2023 Jun 16;3(6):e0001121. doi: 10.1371/journal.pgph.0001121 (PMC10275441; doi:10.1371/journal.pgph.0001121)
Supplement: Attachment — Submitted filename: Response to Reviewers.docx [file pgph.0001121.s002.docx]

**REBUTTAL LETTER**

March 7^th^, 2023

Manuscript reference ID: PGPH-D-22-01451(PLOS Global Public Health)

Title: Design and approval of the nutritional warnings’ policy in Peru: Milestones, key stakeholders, and policy drivers for its approval

Dear editor,

We would like to express our gratitude to you and to the reviewers for the time invested in reading our manuscript. We are thankful for the enriching comments that have helped us to improve the manuscript.

Please find our point-by-point response to all the comments provided below. All the changes have been included in the manuscript and can be identified in the version with track changes uploaded in the Editorial Manager website.

We look forward to hearing from you.

Sincerely,

Francisco Diez-Canseco on behalf of the authors

**Reviewer #1**

1. *Thank you for the opportunity to review this paper. It deals with a very important topic and provides insights into a country’s journey in bringing about the warning labels to discourage unhealthy food consumption and promote informed healthy food choices. This work has some important takeaways for several LAMI countries which are in the process of bringing out FOPL regulations. The methodology is used appears appropriate to document the key milestones, policy drivers and hindrances. I have a few questions and suggestions.*

**Response:** Thank you for your comments and suggestions, that have helped us to improve the manuscript.

2. *The paper in its current form appears too long and difficult to read. This needs to considerably shortened while keeping it to adequate length so as not to lose the flavour of the qualitative work that informs the outcomes. In its current form the paper has too much of narrative and am afraid it will be difficult to engage the readership. I suggest the authors to organise the material to document the key milestones, key drivers, hindrances, how they were overcome. With appropriate sub-heads and second level subheads.*

**Response:** We understand the reviewer’s concern and appreciate the advice to significantly reduce and reorganize the manuscript. The authors agreed to write a manuscript with a detailed results section and chose to submit it to PLOS Global Health, among other reasons, because it allows papers longer than the usual length of ~4,000 words. Considering that this is the first paper that describes the FOPL policy design in Peru, that summarizes 35-hours of interviews with 25 high-profile informants, and that reports a complex multi-stage policy process that lasted almost a decade, we believe that providing a detailed description of some essential aspects of the policy process is crucial. However, following the spirit of the reviewer's suggestion, we have carefully reviewed the results sections to reduce some details, move the Kaleidoscope Model section to a table, and renamed some of the subheadings. In total, we have reduced 1650 words only in the results section.

3. *English language editing is required and the authors if they are not native English speakers may seek help if needed*

**Response:** Two of the manuscript authors are native English speakers and have reviewed the last version of the manuscript.

4. *L54: Indicates that increasing childhood obesity in Peru is the reason for bringing FOPL (Warning labels) regulation. Is it so? Was rising obesity and NCDs among people of all age groups not a reason?*

**Response:** The Law 30021, that included the implementation of FOPL, was called “Law for the Promotion of Healthy Eating for Children and Adolescents” and had a clear focus on this population, in order to reduce the prevalence of NCDs in the future. Rising obesity in children and adolescents was indeed one of the arguments used by advocates of the Law to propose the warnings regulation. Even though they were aware of the increasing obesity in all age groups, they focused on children and adolescents for being a vulnerable population that needed protection. We have added a line in the 2^nd^ paragraph of the Introduction section to clarify that obesity increased in all ages, but especially in children.

5. *L67: delete ‘occur’.*

**Response:** We have edited that sentence in accordance with the suggestion.

6. *L68-70: Not clear ‘Context about the nutritional warnings policy in Peru’ this part is a misfit in the methods section. This should either be in intro or in results.*

**Response:** Thanks for the suggestion. We moved the text to the Introduction section.

Methods

7. *Not clear how the participants were identified? Why 25 Key informants were conducted? Why not 26 or 27 or 40?*

**Response:** An initial group of key informants was identified after reviewing the main policy documents and news as well as having informal conversations with four of them, who helped us to identify the other key players. Then, during each interview, new names emerged and were included in the list of potential informants to be contacted.

Regarding the total number of participants, once we understood the different profiles involved in the design, implementation and monitoring, we defined the final 6 categories of key informants. Based on our previous experience with in-depth qualitative studies and considering the resources available, the team defined to interview 9 policymakers -the most diverse group of informants and with the greatest role in the design and implementation of the policy- and 3 key informants from each of the other 5 profiles (with a total of 24). As in most qualitative studies, we were flexible to include more interviews if we felt that more information was needed and some new key informants were available.

We have clarified this process in the manuscript’s Methods section.

8. *Was media not a key stakeholder group? How about consumer organisations?*

**Response:** Yes, media and industry representatives are together in the 5^th^ profile: “Industry and media representatives”. The informants of consumers organizations are in the 4th profile: “Civil society advocates”. We have provided more details in “Table 1. Description of informants and number or interviews per profile” to better describe our participants’ profiles.

9. *Not clear how masters’ students among researchers were considered a good fit to be called key informants? These were students of which disciplines?*

**Response:** As the manuscript describes, the amount of research around the implementation of the FOPL is scarce in Peru. When searching previous publications or current studies in Peru, as well as mapping key informants, we identified two young researchers that had conducted their master thesis around the FOPL policy. One of them is a sociologist who studied a master in public health in the UK. Her master dissertation was about the arguments used by the food industry during the design and approval of the nutritional warnings policy in Peru. The second researcher is an anthropologist, who did his master in public nutrition in Peru. His dissertation was a comparative analysis about the interference of the food industry in the warning labels policies in Peru, Chile, and Mexico. Both of them had previous experience working in the academia and nowadays continue conducting research in nutrition and policies in public health. We have provided more details about this and other profiles in “Table 1. Description of informants and number or interviews per profile”, in the manuscript’s Methods section.

10. *L128: Not clear if all four researchers were present for every online interview or were they one-to-one speech/discussion events?*

**Response:** Thanks for the comment. Interviews usually involved two or three researchers -with one leading the conversation. We have clarified this in the manuscript.

11. *147-148 triangulation with news and policy documents analysis was an important part of methodology? More clarity is needed on how it was done?*

**Response:** Thanks for the comment. We have clarified in the manuscript’s Methods section that the news and policy documents analysis was a relevant piece of our methodology and better detailed how they were analyzed.

Results

12. *Titles of the tables need to be self-explanatory. They are too shot and colloquial.*

**Response**: We have edited the titles of the tables, providing more information about the table’s contents to ease their comprehension.

13. *Some of the unpublished reports have been included in the scientific events in table-3. How did you source them and were they peer reviewed or were they just studies conducted and not even documented by the ministries/regulators?*

**Response**: We appreciate the observation. Indeed, these documents were studies conducted by the Ministry of Health but they were not peer reviewed and were not published. We knew of these studies from several interviewees who mentioned them, indicating that some key decisions were based on their results (i.e., use of the octagon). We requested access but this was not possible because some of our informants were no longer working on the Ministry of Health and could not find these documents. We have deleted them from Table 3, since PLOS policy does not allow the use of “unpublished data”, and only mentioned them in the text, based on participants’ discourses.

14. *L198: congressmen or congress?*

**Response:** Congressmen is correct. We have corrected this in all the manuscript.

15. *L202-204: how do you say that the congress members were a majority compared to media and industry? Did you mean in the committees that debated the law? The views of the congress outweighed others only because they were a majority?*

**Response:** Thanks for the comment. We reworded the sentence to clarify that the majority of the 120 congressmen supported the Law.

16. *L303-305: This quote is from the regulator/policy maker. Are there any quotes from the opponents of this view from other stakeholder groups.*

**Response**: In this specific topic, most interviewees agreed with this point of view, explaining that this regulation draft was strongly criticized by several stakeholders from different sectors. However, we understand the reviewer’s comment and thereby we have included additional quotes and sentences to show the opponents’ arguments along the Results section.

17. *The narrative in the results section is too long and the reader is likely to lose track of what is being talked about. It only appears to take a predetermined path of showing how the policy makers and politicians with commitment and opinion leaders with a strong will to take materialise the warning labels have maneuvered through tough times. It does not adequately bring out the quotes and views of the opponents.*

**Response:** Thanks for the comment. As mentioned before, we have carefully reviewed the results sections to reduce some details and incorporated some additional quotes to reflect the opponents’ points of view. However, it is true that we focused on the discourses of those in favor of the policy since we aimed to present the achievement of having the policy approved despite the strong opposition faced. Nonetheless, the manuscript details the opponents’ stances in several opportunities, such as in the following lines:

- 262-264: “However, opponents argued that these parameters were based on experts’ opinions and not on an official PAHO document nor scientific evidence”,
- 414-418: “However, opponents at the Cabinet (e.g., Ministry of Economy) argued that the Law did not mention using iconography for the warnings, so that the design could not be mentioned in the draft. Finally, opponents’ argument prevailed, and the published draft did not mention any specific design (see Table 5).”
- And 499-500: “Opponents proposed the traffic lights, GDA or a mix of both, arguing that consumers needed more information (i.e., quantity of nutrients) than solely short messages”

Additionally, and as indicated in response 16, we have added some additional quotes, such as in lines:

- 378-380: ““Trans-fat is the most harmful fat, much more harmful than saturated fat. Therefore, we, of course, in the formulation of our products, we use zero trans-fats.” (Industry representative, IND-01)”
- Or 421-425: ““At some point we wanted to implement it (the traffic lights) in Peru because it seemed to us easier to understand than (the octagons) (…) (Additionally,) you can have High in sugar, yeah, it's High in sugar (but) how high in sugar is it? Is it (a product) (from) someone who made an effort to reformulate the product and instead of 5 grams it has 5.25 or 6 of sugar, versus another one who simply used the High in Sugar label and has 20 grams of sugar per product?” (Food industry representative, IND-02)”

18. *I am not able to connect to the Kaleidoscope Model and the key stages that it talks about. To me table 5 is the key to the results. I appreciate the authors for this. But the same clarity cannot be seen in the narrative.*

**Response**: Thank you for the comment. We have moved previous Table 1 from Introduction to Methods section (now, Table 2) and provided more information to ease the understanding of the Kaleidoscope Model. Additionally, we have incorporated an extra Table (Table 6) to analyze our results based on the hypotheses of the Kaleidoscope Model.

Discussion

19. *The discussion section is a bit weak and ends up repeating the results. Some important points including the how nutrition literacy was enhanced (if at all), how such issues were dealt with in other South American countries, the importance of political will, what were the experiences with industry and media organizations in other countries where warning labels were introduced etc. would help enrich the discussion section.*

**Response**: We appreciate the comment. We have enriched the Discussion section, adding these and some other relevant points in our text.

20. It is not clear what the document search and media coverage data has yielded and how it contributed to the narrative of the paper especially in documenting the timeline and milestones.

**Response:** Thanks for the comment. As mentioned in the comment 11, we have clarified in the manuscript the relevance of analyzing the news and policy documents and how they were used, including how they documented the timeline of milestones.

21. Over all, I suggest the authors to revise the paper to make it more interesting as the paper has some key take ways to offer.

**Response:** Thank you for your feedback. We have improved the manuscript based on your suggestions and we are confident that we have a stronger and clearer paper to be published.

**Reviewer #2:**

1. *It is a well written manuscript describing detailed process followed for approval of the ‘nutritional warnings’ policy in Peru. Authors have provided detailed timelines with policy decisions and action points. The following details may be specified to add more clarity to the manuscript.*

**Response:** Thank you for your comments and suggestions, that have helped us to improve the manuscript.

2. *Line 47-50: Authors are requested to provide reference for processed and ultra processed foods.*

**Response:** We have included a reference in the manuscript.

Methods

3. *Line 106 - 107: It is not clear, how was the sample size of 25 participants calculated? The basis for this should be provided.*

**Response:** Regarding the total number of participants, once we understood the different profiles involved in the design, implementation and monitoring, we defined the final 6 categories of key informants. Based on our previous experience with in-depth qualitative studies and considering the resources available, the team defined to interview 9 policymakers -the most diverse group of informants and with the greatest role in the design and implementation of the policy- and 3 key informants from each of the other 5 profiles (with a total of 24). As in most qualitative studies, we were flexible to include a more interviews if we felt that more information was needed and some new key informants were available.

We have clarified this process in the manuscript’s Methods section.

4. *Lines 104 - 112: The authors selected and interviewed 25 informants who were closely involved in the design, implementation and/or monitoring of the nutritional warnings policy in Peru. Were there additional criteria for selecting the participants/ informants [e.g., years of experience; current job role etc.], if so, the same should be specified.*

**Response:** Thanks for your comment. As you mention, we aim to interview people from different backgrounds that were closely involved in the design, implementation and/or monitoring of the policy. In that sense, the only criteria we defined to select the key informants was to guarantee they played a key role during one or more of these stages or that they had significant knowledge of the policy process because they have studied it (in the case of researchers). During the research, we had a list of the best potential informants that was growing with the suggestions of interviewees, and we always tried to contact first those who were essential for the policy.

5. *Line 107 - 108: The way 'policy makers' and 'politicians' have been defined, were there any overlaps between the two? If so the same should be specified.*

**Response:** Thanks for your question and suggestion. The policymakers are former or current ministers, vice-ministers and civil servants from different levels and Governmental institutions that were or are involved in the design, implementation and/or monitoring of the policy. The politicians are former congressmen closely involved in the design and approval of the Law and its Regulation. We have clarified this in the manuscript and improved the description of all the profiles in Table 2 (now Table 1)..

6. *Table 2: Authors have defined Informants in 5 categories [line 107-109]; Category 2 in table 2, 'Members of Congress' should align with the category specified in text [category 2) politicians; line 108]*

**Response:** Thanks for your comment. We have standardized the categories in the text and the Table 2 (now Table 1).

Results

7. *Line 145 - 146: Please provide reference where the 'implementation and audit process' has been specified*

**Response:** The implementation and audit of the policy will be reported in papers that are still being developed, so no references are available yet. We have corrected this in the manuscript to avoid confusions.

8. Line 207; Extracts of the interviews should be labelled according to the 5 categories of informants defined in lines 107 - 109.

**Response:** Thanks for your suggestion. We have standardized the labels of the quotations according to the initial categories of informants. Moreover, we have standardized the name of the profiles throughout the document.

9. Section 9; The sequence of hypothesis appearing under 'analysis of Kaleidoscope Model' appears randomly and does not follow numerical sequence. If feasible, the hypothesis should appear in numerical sequence for better understanding and clarity.

**Response:** Thank you for the comment. We have incorporated an extra Table in which the hypotheses appear in order to offer more clarity.
